# Acute kidney injury predicts mortality, sarcopenia and postoperative morbidity in myxofibrosarcoma patients undergoing surgical resection

**DOI:** 10.1186/s12957-025-04145-x

**Published:** 2025-12-05

**Authors:** Julian Kylies, Dominik Kylies, Jana K. Striefler, Anna Duprée, Tobias B. Huber, Karl-Heinz Frosch, Matthias Priemel

**Affiliations:** 1 Department of Trauma and Orthopedic Surgery, University Medical Center Hamburg- Eppendorf, Martinistraße 52, Hamburg, 20246 Germany; 2 III. Department of Medicine, University Medical Center Hamburg-Eppendorf, Hamburg, Germany; 3 Hamburg Center for Kidney Health (HCKH), University Medical Center Hamburg-Eppendorf, Hamburg, Germany; 4 Hubertus Wald University Cancer Center Hamburg, University Medical Center Hamburg- Eppendorf, Hamburg, Germany; 5 Department of Oncology, Hematology and Bone Marrow Transplantation with Section Pneumology, Hubertus Wald University Cancer Center, University Medical Center Hamburg- Eppendorf, Hamburg, Germany; 6 Department of General, Visceral and Thoracic Surgery, University Medical Center Hamburg- Eppendorf, Hamburg, Germany; 7 Department of Trauma Surgery, Orthopaedics and Sports Traumatology, BG Klinikum Hamburg, Hamburg, Germany

## Abstract

**Background:**

Myxofibrosarcoma (MFS) is a rare, aggressive soft tissue sarcoma primarily affecting older adults. Despite curative-intent surgery and multimodal therapy, outcomes remain variable. Acute kidney injury (AKI) and sarcopenia are known complications in oncologic care, but their incidence and impact in MFS patients remain unclear.

**Objective:**

To assess the incidence and consequences of AKI in adult MFS patients undergoing curative-intent surgery, and its association with survival, CT-based sarcopenia progression, and postoperative morbidity.

**Methods:**

In this retrospective single-center study, 49 adults with high-grade, N0M0 MFS who underwent curative-intent resection with or without multimodal therapy were included. AKI was defined as a ≥ 0.3 mg/dL increase in serum creatinine. Sarcopenia and adipose tissue loss were assessed via serial CT morphometry scans at the L3 lumbar level. Associations between AKI and clinical outcomes (survival, function, length of hospital stay, surgical site infection) were evaluated using regression and survival analyses.

**Results:**

AKI occurred in 38.8% of patients and independently predicted decreased overall survival (HR 6.73; *p* = 0.0005). AKI was associated with accelerated loss of muscle tissue and visceral fat (all *p* < 0.001), functional decline (ECOG, *p* = 0.0078), longer hospitalization (*p* < 0.001), and increased wound infections (52.6% vs. 10.0%; *p* < 0.0001).

**Conclusions:**

This is the first study to identify AKI as an independent predictor of mortality and sarcopenia in surgically treated MFS patients. CT morphometry may aid in early risk stratification, while renal monitoring offers a potential target for improving outcomes through nephroprotective strategies.

**Supplementary Information:**

The online version contains supplementary material available at 10.1186/s12957-025-04145-x.

## Introduction

 Myxofibrosarcoma (MFS) is a rare, aggressive fibroblast-derived sarcoma with infiltrative growth and high local recurrence rates [1, 2]. Surgical resection combined with multimodal therapy remains the mainstay of treatment, yet postoperative morbidity is substantial and long-term outcomes remain variable [3–5].

Acute kidney injury (AKI) is an increasingly recognized complication in surgical oncology and has been linked to decreased survival, diminished quality of life, increased morbidity, prolonged hospitalization, and impaired treatment tolerance [6–9]. Despite this, the incidence and prognostic significance of AKI remain virtually unstudied in adult MFS patients, a particularly vulnerable group due to their age, comorbidity burden, and frequent exposure to nephrotoxic chemotherapy or extensive surgery.

Sarcopenia, characterized by progressive loss of skeletal muscle and visceral adipose tissue, is a well-established predictor of frailty, poor treatment tolerance, and mortality across a broad range of tumor entities [10–16]. Reliable clinical assessment of sarcopenia remains difficult, as existing tools are often subjective and resource-intensive, limiting routine use. CT-based body composition analysis (CT-morphometry) has therefore emerged as a reproducible, objective method to quantify muscle mass and quality. Metrics such as the skeletal muscle index (SMI), paraspinal muscle index (PSMI), psoas muscle index (PMI), and skeletal muscle density (SMD) provide standardized assessments of muscle status, while visceral adipose tissue (VAT) serves as a marker of metabolic reserve and has been linked to treatment tolerance and survival in cancer patients [16–19].

Beyond its impact on surgical and oncologic outcomes, sarcopenia has also been linked to kidney dysfunction in both experimental and clinical studies [20–24]. Impaired muscle mass may not only result from renal impairment but also accelerate its progression through shared inflammatory and metabolic pathways [25–28]. In surgical patients, sarcopenia increases the risk of postoperative kidney injury and worsens survival, suggesting a bidirectional relationship between muscle wasting and renal vulnerability [29].

To optimize the care of adult patients with myxofibrosarcoma treated with curative-intent surgery and multimodal therapy, we investigated the incidence and prognostic significance of AKI in adult myxofibrosarcoma patients undergoing curative-intent surgery. We assessed whether AKI independently predicts survival, complications, functional decline, and sarcopenia progression, to better understand the drivers of prognosis in this elderly, high-risk cohort.

## Materials and methods

### Study design and patient selection

This retrospective study was approved by the local ethics committee (ID: 2025-300576-WF) and conducted in accordance with the principles of the Declaration of Helsinki. Owing to the anonymized and retrospective nature of the study, the requirement for informed consent was waived.

**Fig. 1:**
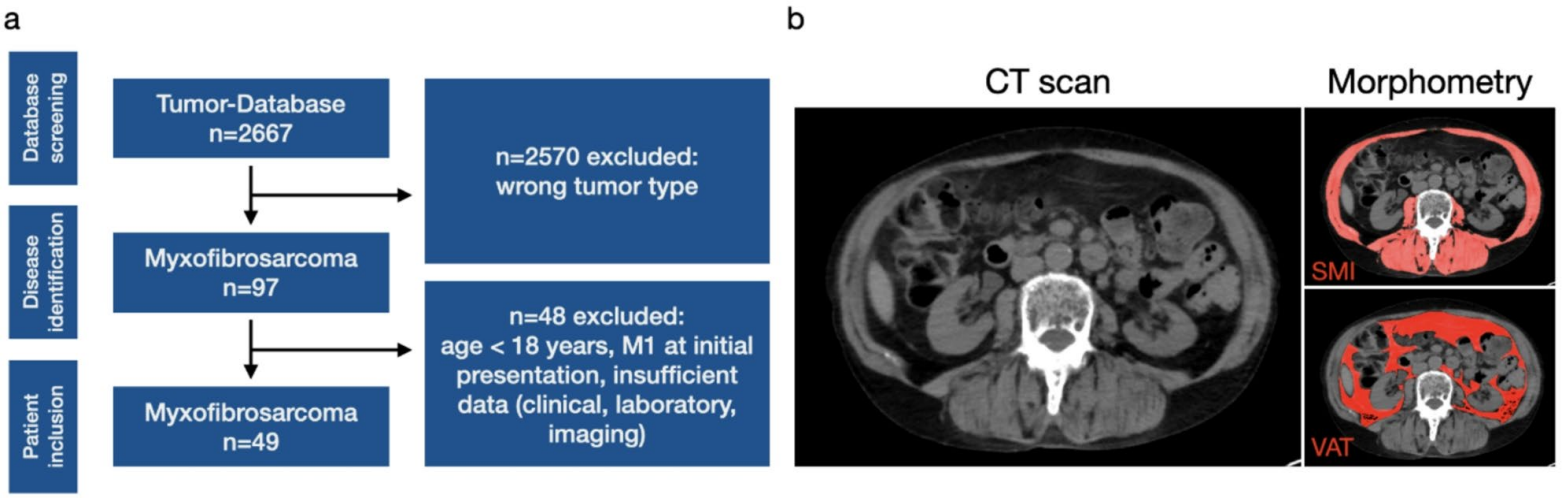
Myxofibrosarcoma patient selection and illustration of CT morphometry. **a** A systematic screening of our tumor database identified 2667 tumor patients. After excluding 2570 patients with non-myxofibrosarcoma tumors, we identified a total of 97 myxofibrosarcoma patients for further eligibility screening. 48 myxofibrosarcoma patients were excluded due to an age < 18 years, metastatic disease at initial presentation or missing laboratory, clinical or imaging data. 49 myxofibrosarcoma patients were included in our final analysis. **b** Exemplary demonstration of CT-morphometrical assessment of SMI and VAT

49 patients with a histologically confirmed diagnosis of high-grade myxofibrosarcoma (MFS) who underwent surgical resection between 2010 and 2024 and did not meet exclusion criteria were included in this analysis. Other inclusion criteria were a baseline TNM stage of N0M0, high-quality CT imaging with adequate visualization of the L3 vertebral level at least two consecutive time points (one obtained prior to surgery and another acquired at a minimum of 12 months postoperatively), complete clinical and laboratory data including ECOG performance status, resection margin status, TNM classification and laboratory data including kidney function assessments available at both imaging time points. Additional collected variables included surgical site infection rate and length of hospital stay following surgery. Exclusion criteria included age < 18 years, metastatic disease at baseline as well as incomplete clinical, laboratory or imaging data (specifically if less than two CT scans were available at the time points discussed above). Furthermore, patients were also excluded if the available CT imaging quality was not sufficient for morphometry analysis (i.e. due to artefacts). Laboratory and clinical parameters were extracted from electronic patient file at the first and last CT-time point. The patient selection is illustrated in Fig. 1a.

### Analysis of CT-morphometrics

CT-morphometrics was assessed using Fiji imaging software (Version 2.3.0/1.53q, Max Planck Institute of Molecular Cell Biology and Genetics, Dresden, Germany) with semi-automated thresholding, as previously described. All CT-morphometrics was assessed at the L3 vertebral 0level. Skeletal muscle areas were quantitatively assessed using standardized Hounsfield unit (HU) thresholds (range: -29 to + 150 HU for muscle parameters, -190 to -30 HU for VAT) and normalized to patient height (cm²/m²). The following morphometric parameters were assessed: SMI, PSMI, PMI and VAT. SMD, an additional parameter for muscle quality was assessed by measuring the average HU of the paraspinal musculature Fig. 1b.

### Statistical analysis

The statistical analyses as well as generation of graphical illustrations were performed using GraphPad Prism (version 10.4.1) and RStudio (R version 4.4.2). Longitudinal comparisons of CT-morphometric parameters across time points were performed using the Wilcoxon matched-pairs signed rank test. Pairwise comparisons of CT-morphometrics and laboratory parameters between the kidney injury and non-kidney injury groups were performed using the Mann–Whitney U test. Survival was assessed using Kaplan–Meier analysis [30] with Log-Rank (Mantel–Cox) tests and Cox proportional hazards regression models (both univariate and multivariate), with model comparison conducted using the Likelihood Ratio Test. For survival analyses, only patients between 2010 and 2020 were included to ensure a robust follow-up of at least 5 years. All continuous data are reported as mean ± standard deviation (SD) unless otherwise specified. All statistical tests were two-tailed, p-values < 0.05 were interpreted as statistically significant.

## Results

### Patient identification and -characteristics

A systematic screening of our tumor database was performed. We initially identified 2667 patients and excluded 2570 with non-MFS tumor-entities, identifying 97 total MFS-patients. After carefully checking and employing in- and exclusion criteria as described in the materials and methods section, 48 further MFS patients were excluded. The remaining 49 MFS patients (18 female, 31 male) were included in the final analysis (Fig. 1a). Patient characteristics are listed in Table 1. 19 Patients (38.8%) developed kidney injury throughout the course of this study. Kidney injury was defined as an increase in serum creatinine of ≥ 0.3 mg/dl over the study period. The overall mean age was 72 ± 12.9 years, there was no significant difference in age between patients with and without kidney injury (71 ± 13.0 vs. 73 ± 13.0 years, *p* = 0.79) (Supplementary Fig. 1a). The mean baseline serum creatinine was 0.9 ± 0.18 mg/dl for all patients and there were no significant differences in serum creatinine at baseline between kidney injury- and non-kidney injury-MFS-patients (0.9 ± 0.14 vs. 1.0 ± 0.19 mg/dl, *p* = 0.08) (Supplementary Fig. 1b). 44 out of 49 total MFS patients had an eGFR ≥ 60 ml/min. The median ECOG-score at baseline was 0.5 (range = 2) for all MFS-patients and did not differ between MFS-patients developing kidney injury (median ECOG 0.5, range = 2) and those without kidney injury (median ECOG 0.5, range = 2) (*p* = 0.96, Supplementary Fig. 1c). 29 MFS-patients (59.2%) developed local recurrent disease over the course of our study with comparable rates in kidney injury- and non-kidney injury patients (*n* = 11/19 (57.9%) vs. *n* = 18/30 (60.0%), *p* > 0.99, Supplementary Fig. 1d). More MFS patients in the non-kidney injury group had surgery only (without radiation or chemotherapy) compared to the kidney injury group (*n* = 16 (53.3%) vs. *n* = 4 (21.1%), *p* = 0.037 Supplementary Fig. 1e. While no statistically significant trend was observed in the rates of added-radiotherapy between kidney injury and non-kidney injury MFS-patients (*n* = 3 (15.8%) vs. *n* = 11 (36.7%), *p* = 0.19, Supplementary Fig. 1f), significantly more MFS-patients in the kidney injury-group received chemotherapy compared to those without kidney injury (*n* = 13 (68.4%) vs. *n* = 6 (20%), *p* = 0.001) (Supplementary Fig. 1g).

**Table 1:** Patient characteristics

	Overall	No kidney injury	kidney injury
Patients	49	30	19
Age	72 (± 12.9)	73 (± 13.0)	71 (± 13.0)
Creatinine (mg/dl)	0.9 (± 0.18)	1.0 (± 0.19)	0.9 (± 0.14)
eGFR (ml/min)			
≥ 60	44	26	18
45–59	4	4	0
30–44	1	0	1
15–29	0	0	0
< 15	0	0	0
ECOG at baseline	0.5 (range 2)	0.5 (range 2)	0.5 (range 2)
Local tumor recurrence (LR)	29 (59.2%)	18 (60.0%)	11 (57.9%)
Surgery only	20 (40.8%)	16 (53.3%)	4 (21.1%)
+ Radiation	14 (28.6%)	11 (36.7%)	3 (15.8%)
+ Chemotherapy	19 (38.8%)	6 (20%)	13 (68.4%)

Age and Creatinine were displayed as mean with SD, ECOG at baseline was reported as median with range

### Impact of kidney injury on patient survival

**Fig. 2:**
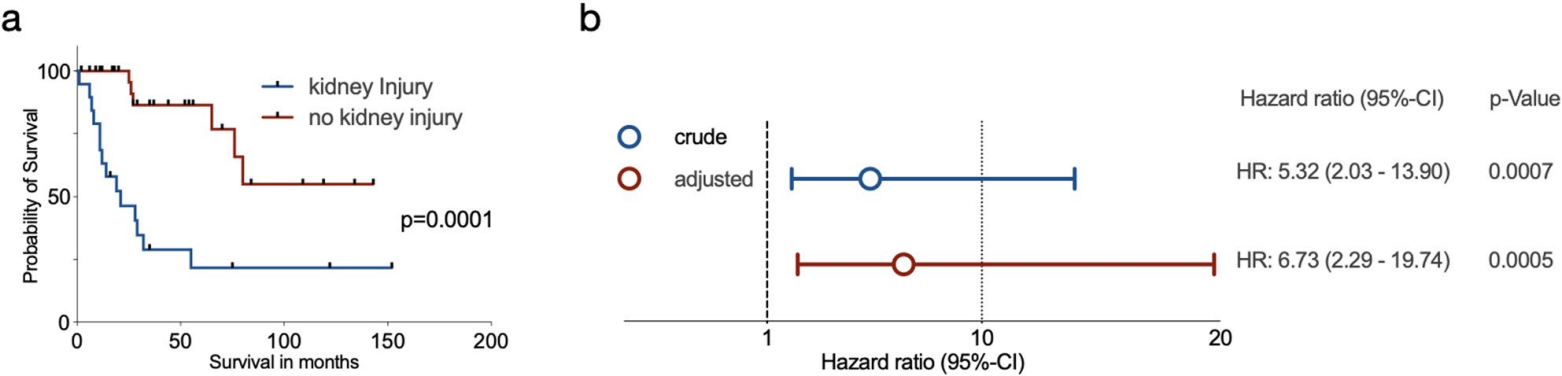
Kaplan Meier Survival analysis and Cox proportional Hazard Ratio. **a** Kaplan-Meier analysis shows a significantly decreased survival probability in myxofibrosarcoma patients who developed kidney injury. **b** Hazard ratio calculation for kidney injury using a Cox proportional hazards regression model. In both the crude as well as the adjusted analysis, kidney injury was associated with a significantly increased hazard of mortality

Kaplan–Meier survival analyses as well as uni- and multivariate Cox proportional hazards regression models (Table 2) were conducted to delineate the clinical and prognostic impact of kidney injury in patients with MFS. Kaplan-Meier survival analysis revealed a significant difference in survival between MFS-patients with and without kidney injury (*p* < 0001). The median survival in the kidney injury group was 21.00 months, whereas the median survival time for the non-kidney injury group was not estimable within follow-up as less than 50% of MFS-patients without kidney injury died within our observation period (Fig. 2a).

**Table 2:** Uni- and multivariate Cox proportional hazards regression analysis

Univariate			
Variable	HR	95% CI	*p*-value
Kidney injury	5.32	2.03–13.90	0.0007
Age	1.02	0.98–1.06	0.3289
Local recurrence	2.97	1.20–7.34	0.0183
Baseline ECOG	0.84	0.43–1.63	0.6035
Tumor diameter	0.97	0.84–1.13	0.7049

Multivariate			
Variable	HR	95% CI	*p*-value
Kidney injury	6.73	2.29–19.74	0.0005
Age	1.03	0.99–1.07	0.1737
Local recurrence	2.75	0.98–7.77	0.0555
Baseline ECOG	0.84	0.40–1.74	0.6390
Tumor diameter	1.02	0.85–1.21	0.8611

In the univariate Cox regression analysis, the occurrence of kidney injury was significantly associated with increased mortality in patients with MFS (HR: 5.32; 95% CI: 2.03–13.90; *p* = 0.0007). In the multivariate analysis adjusting for age, local recurrence, baseline ECOG status, and tumor diameter, kidney injury remained an independent predictor of overall survival (HR: 6.73; 95% CI: 2.29–19.74; *p* = 0.0005, Fig. 2b). A trend towards worse prognosis was also observed for local recurrence in the multivariate analysis (HR: 2.75; 95% CI: 0.98–7.77; *p* = 0.0555), although this did not reach statistical significance. These findings underscore the prognostic importance of kidney injury in MFS-patients, highlighting kidney injury as an independent risk factor for increased mortality in this cohort.

### Changes in sequentially assessed CT-morphometrics in MFS patients with kidney injury

We next analyzed the changes in sequentially assessed CT-morphometrics in MFS patients with kidney injury.

At baseline, CT-morphometrics displayed no difference between MFS-patients developing kidney injury compared to those without kidney injury (SMI 46.13 ± 5.38 cm^2^/m^2^ vs. 44.35 ± 6.62 cm^2^/m^2^, *p* = 0.28, Supplementary Fig. 2a, PSMI 17.25 ± 1.15 cm^2^/m^2^ vs. 17.26 ± 1.42 cm^2^/m^2^, *p* = 0.82, Supplementary Fig. 2b, PMI 2.57 ± 0.45 cm^2^/m^2^ vs. 2.60 ± 0.61 cm^2^/m^2^, *p* = 0.93, Supplementary Fig. 2c, VAT 82.23 ± 7.83 cm^2^/m^2^ vs. 80.74 ± 6.87 cm^2^/m^2^, *p* = 0.39, Supplementary Fig. 2d, SMD 42.05 ± 4.48 HU vs. 42.80 ± 3.55 HU, *p* = 0.39, Supplementary Fig. 2e in kidney injury vs. no kidney injury, respectively).

**Fig. 3:**
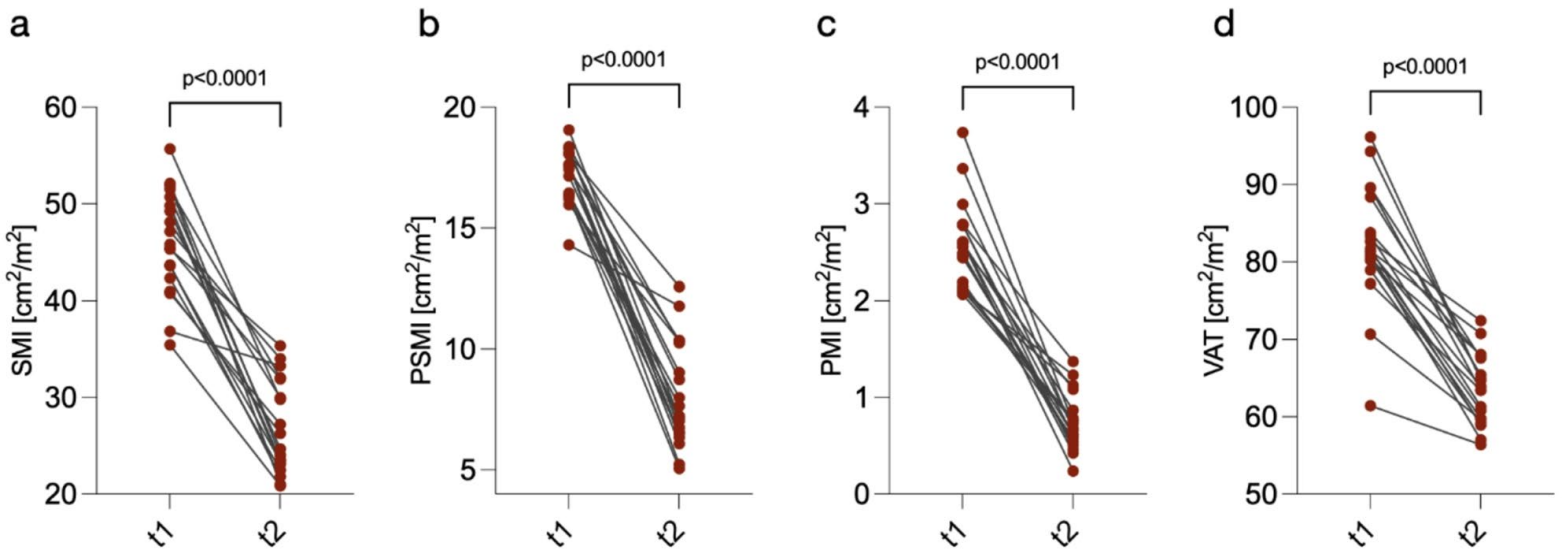
Analysis of sequential CT-morphometry in myxofibrosarcoma patients developing kidney injury. **a** Patients developing kidney injury exhibit significant decreases in SMI over the course of the study between t1 and t2. **b** PSMI declined significantly kidney injury patients between t1 and t2. **c** Myxofibrosarcoma patients with kidney injury showed significant reductions in PMI between t1 and t2. **d** VAT decreased significantly in myxofibrosarcoma patients with kidney injury between t1 and t2

In MFS patients developing kidney injury the SMI decreased from 46.13 ± 5.38 cm^2^/m^2^ at baseline (t1) to 26.49 ± 5.12 cm^2^/m^2^ at follow up (t2) (*p* < 0.0001, Fig. 3a). Similarly, MFS patients with kidney injury developed a significantly higher loss in PSMI between t1 and t2 (17.25 ± 1.15 cm^2^/m^2^ vs. 8.04 ± 2.16 cm^2^/m^2^, *p* < 0.0001, Fig. 3b). Like SMI and PSMI, PMI also significantly decreased over the course of the study in MFS patients with kidney injury with a baseline value at t1 of 2.57 ± 0.45 cm^2^/m^2^ decreased to 0.74 ± 0.29 cm^2^/m^2^ at t2 (*p* < 0.0001, Fig. 3c). In addition to muscle parameters, VAT, a marker of abdominal adipose tissue also decreased significantly over the course of the study between t1 and t2 in MFS patients developing kidney injury (82.23 ± 7.83 cm^2^/m^2^ vs. 63.46 ± 4.53 cm^2^/m^2^, *p* < 0.0001, Fig. 3d). While there was a trend in decline in SMD between t1 and t2 in MFS patients with kidney injury, it did not reach statistical significance (42.05 ± 4.48 HU vs. 40.04 ± 5.39 HU, *p* = 0.23, Supplementary Fig. 3a).

Overall, these findings highlight a progressive loss in muscle mass and visceral adipose tissue in MFS patients developing kidney injury over the course of the disease.

### Comparisons in CT-morphometrics trajectory of MFS patients with and without kidney injury

Subsequently, we compared the changes in CT-morphometrics of MFS patients developing kidney injury to those without kidney injury during the course of the study.

**Fig. 4:**
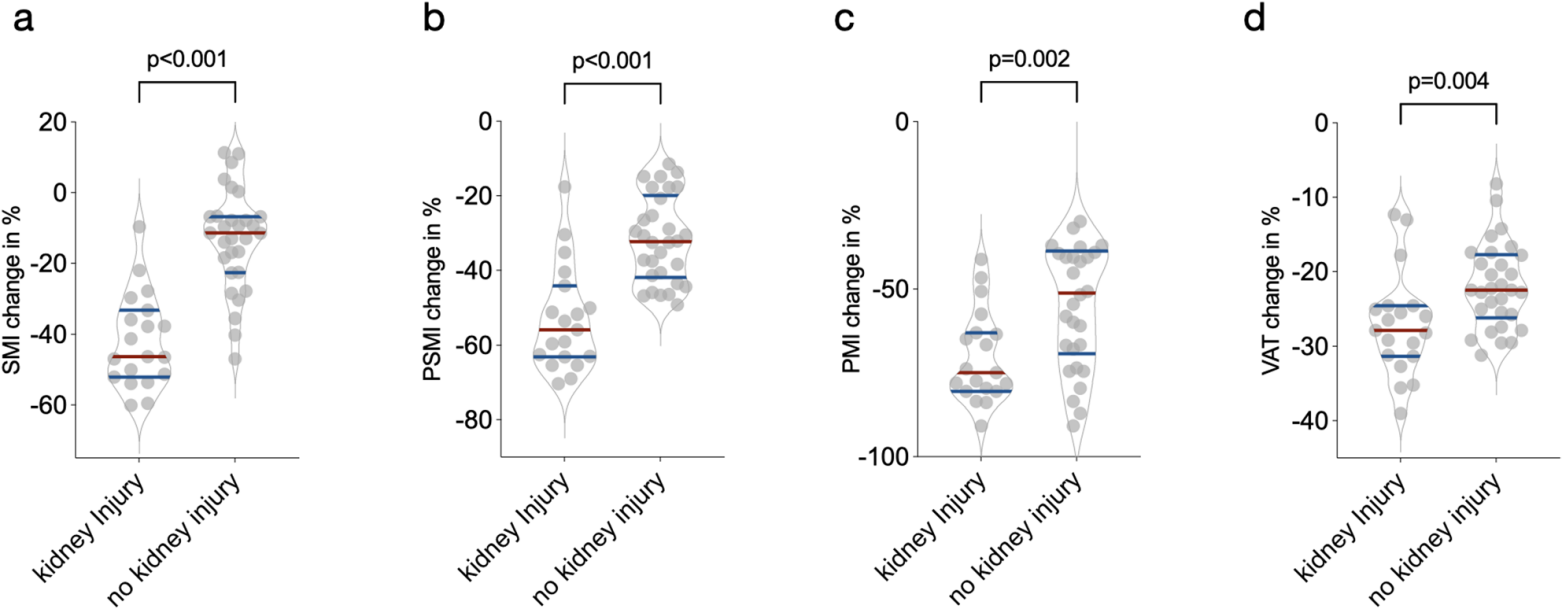
Comparative Analysis of CT-morphometry in MFS patients with and without kidney injury. **a** Patients with MFS who developed kidney injury showed a significantly more pronounced negative change in SMI compared to those who did not develop kidney injury. **b** Among MFS patients, those who experienced kidney injury exhibited a significantly greater decline in PSMI than those without kidney injury. **c** The reduction in PMI was significantly more pronounced in MFS patients who developed kidney injury compared to those who did not. **d** Patients developing kidney injury showed a significantly higher loss of VAT compared to those without kidney injury

MFS-patients developing kidney injury over the course of the study exhibited a significantly greater reduction in SMI, with a mean decline of -41.85 ± 13.27% compared to -13.25 ± 14.29% in the non-kidney injury group (*p* < 0.001, Fig. 4a). PSMI also displayed a significantly greater decrease in the kidney injury group compared to the non-kidney injury group (-53.02 ± 14.08% vs. -31.68 ± 11.56%, *p* < 0.001, Fig. 4b). Similarly, The PMI decreased significantly more in the kidney injury group with − 70.21 ± 13.69% compared to the non-kidney injury group with − 49.71 ± 28.99% (*p* = 0.002, Fig. 4c). Additionally, VAT decreased significantly more in the kidney injury group compared to the non-kidney injury group (-27.1 ± 6.99% vs. -21.91 ± 5.67%, *p* = 0.004, Fig. 4d). SMD showed a trend towards a more pronounced decline in the kidney injury group compared to the non-kidney injury group that did not reach statistical significance (-3.83 ± 16.0% vs. -2.7 ± 25.9, *p* > 0.99, Supplementary Fig. 3b). Overall, these results suggest that the development of kidney injury in patients with MFS is associated with significantly greater reductions in parameters of skeletal muscle and visceral adipose tissue measured by CT-morphometry compared to MFS-patients without kidney injury.

### Impact of acute kidney injury on functional status, length of hospital stays and wound infections

**Fig. 5:**
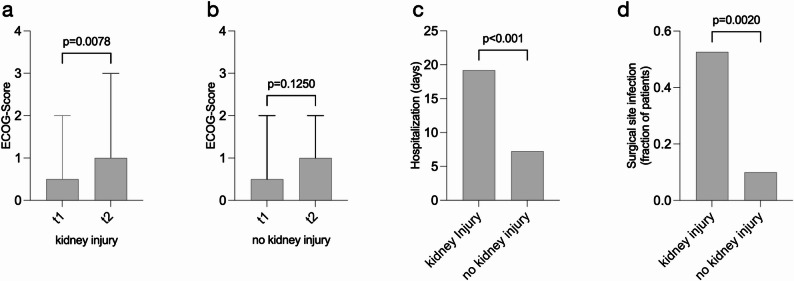
Impact of acute kidney injury on functional status, length of hospital stays and wound infections (**a**) MFS-patients with kidney injury developed a significant increase in ECOG score between t1 and t2. **b** MFS-patients without kidney injury did not experience an increase in ECOG score during the duration of the study. **c** Patients with kidney injury displayed a significantly prolonged hospitalization time compared to those without kidney injury. **d** In MFS-patients, kidney injury was associated with significantly more wound infections

Ultimately, we analyzed the impact of kidney injury in MFS-patients on functional status as measured by ECOG score, length of hospital stays and surgical site infection rates. MFS-patients developing kidney injury showed a decline in functional status as reflected by a significant increase in median ECOG score between t1 and t2 (median ECOG 0.50, range = 2 vs. 1.0, range = 3, *p* = 0.0078, Fig. 5a). In comparison, MFS-patients not developing kidney injury did not show a significant functional decline as reflected ECOG scores over the course of the study (median ECOG t1 vs. t2: 0.5, range = 2 vs. 1.0, range 2, *p* = 0.125, Fig. 5b). MFS patients developing kidney injury also displayed a significantly increased length in hospital stay compared to those without kidney injury (17.74 ± 7.63 days vs. 7.27 ± 2.27 days, *p* < 0.001, Fig. 5c). Notably, patients without primary surgical closure (e.g., vacuum-assisted wound therapy) were excluded from this analysis to avoid confounding due to prolonged hospitalization. The rate of wound infections was also significantly higher in MFS-patients with kidney injury compared to MFS-patients without kidney injury (52.63% vs. 10.0%, *p* = 002, Fig. 5d. Together, these findings highlight the implications on increased overall morbidity and clinical complications of kidney injury in MFS-patients.

## Discussion

Myxofibrosarcoma (MFS) is typically managed with wide resection, often supplemented by radiotherapy or cytotoxic chemotherapy in an elderly, comorbid population. To our knowledge, this is the first study to systematically investigate AKI in adult patients with MFS undergoing curative-intent surgery. Our findings identify AKI as a frequent and clinically significant complication in this setting, with profound implications for both surgical and functional outcomes. Nearly 40% of MFS patients in our cohort developed AKI during the disease course, a remarkably high incidence given the limited prior recognition of renal complications in this population. Importantly, the occurrence of AKI was independently associated with a more than six-fold increase in mortality, even after adjustment for key clinical variables including age, ECOG status, local recurrence, and tumor diameter, suggesting AKI as a key clinical determinant of survival in MFS patients.

Beyond its impact on overall survival, AKI was closely linked to a cascade of adverse outcomes including accelerated sarcopenia, functional deterioration, and increased postoperative morbidity. Longitudinal CT-morphometric analyses revealed that patients with AKI experienced significantly greater declines in skeletal muscle and adipose tissue parameters (SMI, PSMI, PMI, and VAT), compared to those with preserved kidney function. These radiographic changes were paralleled by a worsening of ECOG performance status, prolonged hospital stays, and a five-fold higher rate of surgical site infections.

Taken together, these findings support a bidirectional relationship between kidney dysfunction and clinical and metabolic decline. On the one hand, kidney dysfunction may directly contribute to catabolism through several interrelated mechanisms. Systemic inflammation and oxidative stress are well-established drivers of muscle breakdown, while the accumulation of uremic toxins can impair protein synthesis and promote muscle wasting, also know as “uremic catabolism”. In addition, hormonal dysregulation, including insulin resistance and reduced anabolic signaling, further accelerates the loss of muscle and adipose tissue mass. Off note, inter-organ crosstalks between kidney and muscle have been identified under both experimental and clinical conditions, linking impaired kidney function to progressive muscle wasting [27]. Interestingly, at least under experimental conditions, kidney-induced muscle wasting could pharmacologically be addressed, thereby, at least theoretically providing promising options for future research and potentially therapeutic approaches. Overall, these pathophysiological changes provide a plausible explanation for the more pronounced sarcopenic trajectory observed in MFS patients with kidney injury [31–33]. On the other hand, baseline sarcopenia may predispose patients to renal injury by masking early changes in creatinine and compromising physiological reserve during chemotherapy or surgical stress [34–36]. This interplay reinforces the concept of a kidney–muscle axis [27] in oncologic surgery, particularly relevant in the older, multimorbid MFS population.

Our results underscore the need to integrate renal risk assessment into standard perioperative sarcoma care. Routine renal monitoring, including serial serum creatinine and eGFR measurements, should be implemented in patients undergoing chemotherapy or major surgery. In addition, systematic CT-based body composition analysis (SMI, VAT) may help identify individuals at highest risk of AKI and sarcopenia. Such patients could benefit from early nephroprotective strategies, such as adjusted chemotherapy dosing, optimized hydration protocols, and avoidance of nephrotoxic agents, as well as targeted prehabilitation, nutritional support, and intensified postoperative surveillance. By embedding these measures into routine care, clinicians may help break the cycle of frailty, renal stress, and clinical deterioration, thereby improving resilience to treatment and long-term outcomes [37, 38].

While our study provides novel insights, several limitations must be acknowledged. The retrospective, single-center design and modest sample size limit generalizability, and residual confounding cannot be excluded. In particular, the marked imbalance in chemotherapy exposure between groups (68.4% vs. 20%) may have influenced both the occurrence of kidney injury and the progression of sarcopenia and should therefore be considered a potential confounder. Moreover, the use of serum creatinine to define AKI may underestimate renal impairment in sarcopenic individuals. Nonetheless, our consistent findings across clinical, functional, and radiographic domains strengthen the validity of our conclusions.

In summary, AKI is a common and clinically relevant complication in adult patients with myxofibrosarcoma undergoing surgery, independently predicting mortality, sarcopenia progression, and postoperative morbidity. These findings position AKI as a clinically highly relevant and potentially modifiable risk factor with practical implications for surgical oncology, and highlight the potential of CT morphometry and renal surveillance to inform risk-adapted, multidisciplinary management strategies in high-risk soft tissue sarcoma patients.

## Supplementary Information

Supplementary Material 1.

## Data Availability

Data can be made available upon request.
