# Supplementary material for: Acute kidney injury predicts mortality, sarcopenia and postoperative morbidity in myxofibrosarcoma patients undergoing surgical resection
Source: World J Surg Oncol. 2025 Dec 5;23:451. doi: 10.1186/s12957-025-04145-x (PMC12690947; doi:10.1186/s12957-025-04145-x)
Supplement: Supplementary file 1 — Supplementary Material 1. [file 12957_2025_4145_MOESM1_ESM.docx]

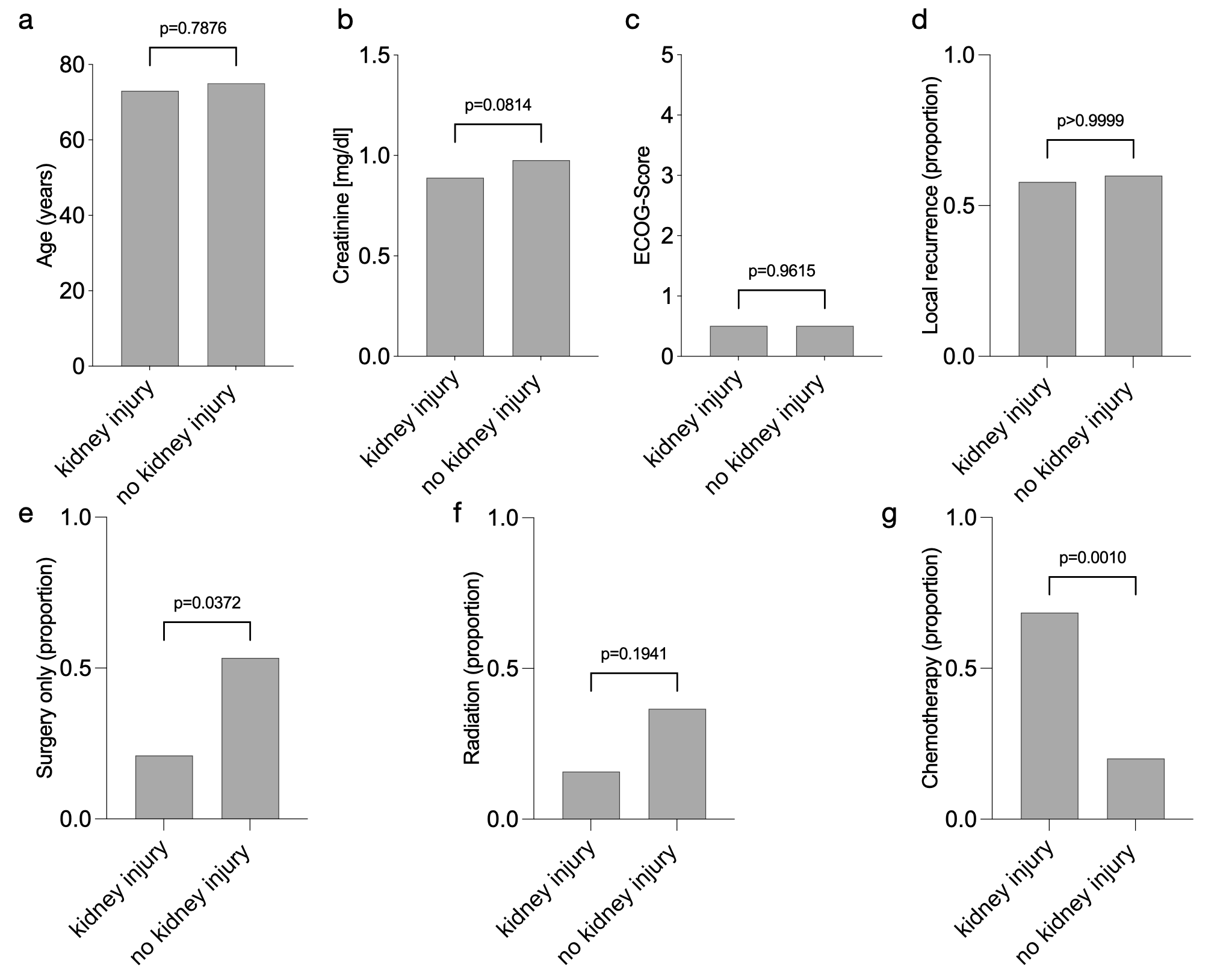


**Supplementary Figure Fig. 1 | Comparison of clinical parameters in MFS patients developing kidney injury compared to MFS patients without kidney injury. a.** No significant difference in age was observed between MFS patients who developed kidney injury and those who did not. **b.** Baseline creatinine levels did not significantly differ between patients with and without kidney injury. **c.** ECOG performance status showed no statistically significant variation between the two groups. **d.** The incidence of local recurrence was comparable between MFS patients with kidney injury and those without. **e.** The proportion of patients managed with surgery only was significantly lower among those who developed kidney injury. **f.** The use of radiotherapy did not differ significantly between patients with and without kidney injury. **g.** A significantly higher proportion of patients with kidney injury received chemotherapy compared to those without.


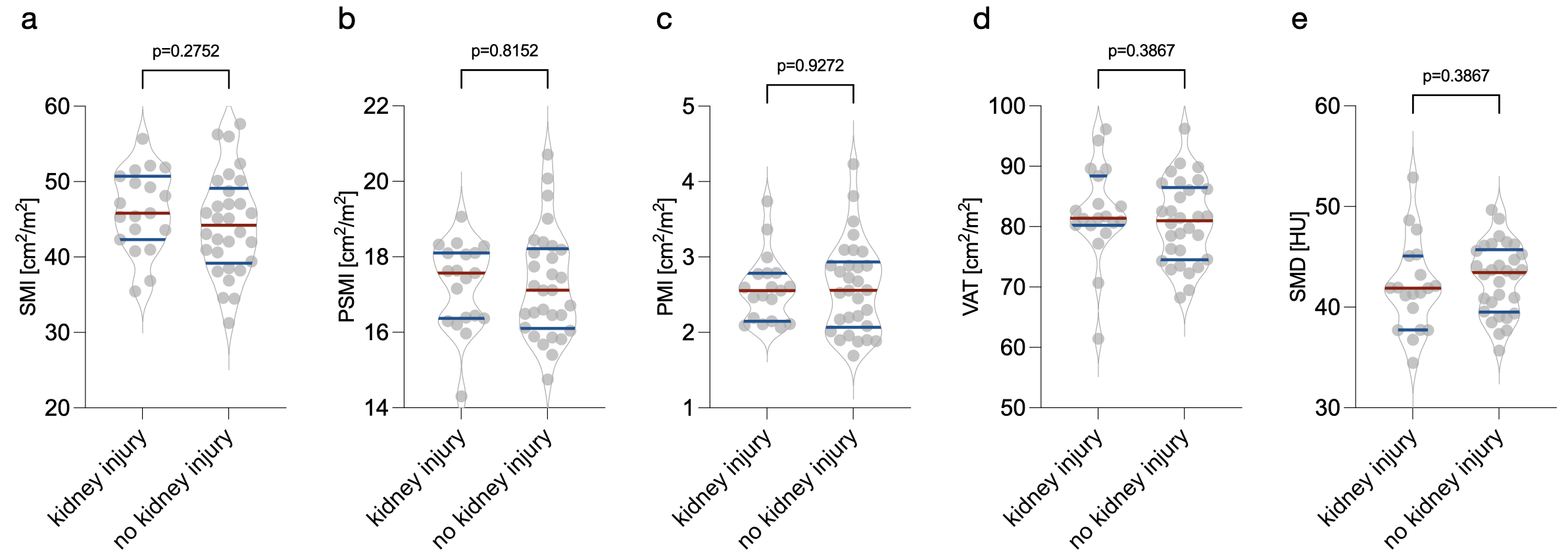


**Supplementary Figure 2 | Baseline CT-morphometry in MFS patients with and without kidney injury.** **a.** No significant differences in SMI were observed between MFS patients with and without kidney injury. **b.** PSMI did not significantly differ between MFS patients with and without kidney injury. **c.** There was no statistically significant difference in PMI between MFS patients with and without kidney injury. **d.** Analysis revealed no significant difference in VAT between MFS patients with kidney injury and those without. **e.** MFS patients with and without kidney injury showed no significant difference in levels of SMD.


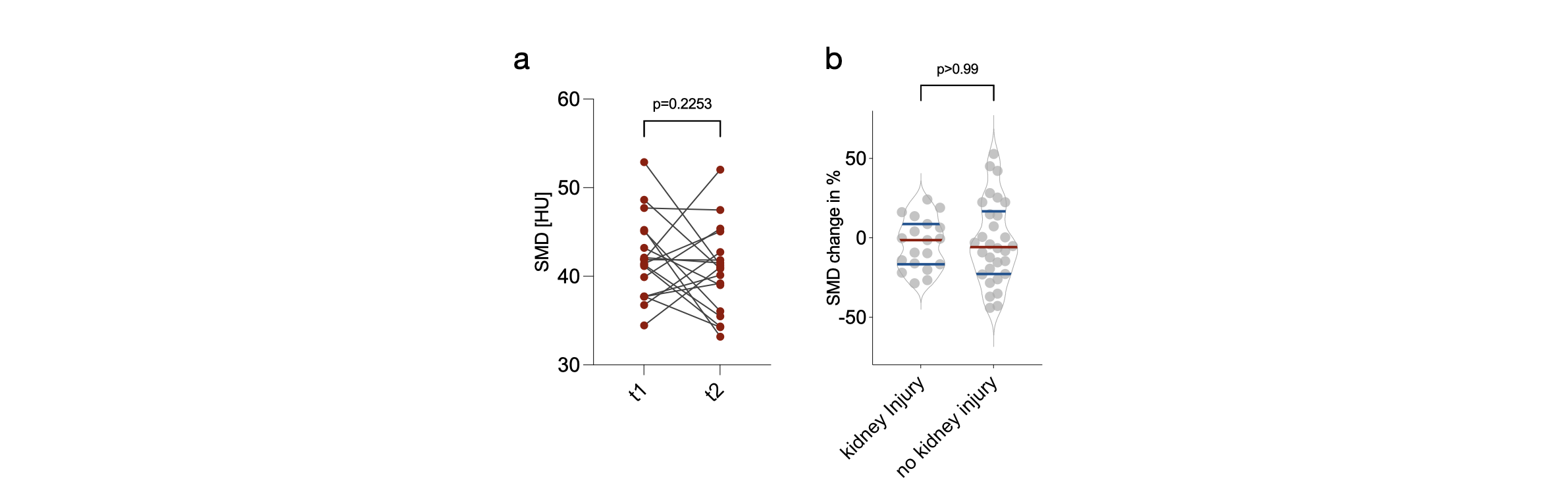


**Supplementary Figure Fig. 3 | Additional CT-morphometrics. a.** No statistically significant difference in sequential assessment of SMD in MFS patients developing kidney injury was found between t1 and t2 **b.** Analysis of SMD in MFD patients with and without kidney injury revealed no statistically significant difference between the two groups.
